# SGLT2 inhibition improves PI3Kα inhibitor–induced hyperglycemia: findings from preclinical animal models and from patients in the BYLieve and SOLAR-1 trials

**DOI:** 10.1007/s10549-024-07405-8

**Published:** 2024-08-23

**Authors:** Manuel Ruiz Borrego, Yen-Shen Lu, Felipe Reyes-Cosmelli, Yeon Hee Park, Toshinari Yamashita, Joanne Chiu, Mario Airoldi, Nicholas Turner, Luis Fein, Farhat Ghaznawi, Jyotika Singh, Kristyn Pantoja, Christian Schnell, Murat Akdere, Stephen Chia

**Affiliations:** 1 Hospital Universitario Virgen del Rocío de Sevilla, Seville, Spain; 2 National Taiwan University Hospital, Taipei, Taiwan; 3 Instituto Oncológico Fundación Arturo López Pérez, Santiago, Chile; 4 Samsung Medical Center, Sungkyunkwan University School of Medicine, Seoul, South Korea; 5 Kanagawa Cancer Center, Yokohama, Japan; 6 Queen Mary Hospital, Pok Fu Lam, Hong Kong; 7 Azienda Ospedaliero Universitaria Città della Salute e della Scienza di Torino, Turin, Italy; 8 The Royal Marsden NHS Foundation Trust, London, UK; 9 Instituto de Oncología de Rosario, Santa Fe, Argentina; 10 Novartis Pharmaceuticals Corporation, East Hanover, NJ USA; 11 Novartis Healthcare Private Ltd, Hyderabad, India; 12 Novartis Pharmaceuticals Corporation, Cambridge, MA USA; 13 Novartis Pharma AG, Basel, Switzerland; 14 BC Cancer, Vancouver, BC Canada

**Keywords:** Alpelisib, Hyperglycemia, SGLT2 inhibitor, HR + /HER2 −, Advanced breast cancer

## Abstract

**Purpose:**

Alpelisib plus fulvestrant demonstrated a significant progression-free survival benefit versus fulvestrant in patients with *PIK3CA-*mutated HR+ /HER2− advanced breast cancer (ABC) (SOLAR-1). Hyperglycemia, an on-target adverse effect of PI3Kα inhibition, can lead to dose modifications, potentially impacting alpelisib efficacy. We report data from preclinical models and two clinical trials (SOLAR-1 and BYLieve) on Sodium glucose cotransporter 2 inhibitor (SGLT2i) use to improve PI3Kα inhibitor–associated hyperglycemia.

**Methods:**

Healthy Brown Norway (BN), mild diabetic Zucker diabetic fatty (ZDF), and Rat1-myr-p110α/HBRX3077 tumor–bearing nude rats treated with alpelisib were analyzed for glucose and insulin control with metformin and dapagliflozin (SGLT2i) and alpelisib efficacy. Hyperglycemia adverse events (AEs) were compared between patients receiving SGLT2i with alpelisib (n = 19) and a propensity score–matched cohort not receiving SGLT2i (n = 74) in both trials.

**Results:**

Dapagliflozin and metformin in BN and ZDF rats treated with alpelisib normalized blood glucose and reduced insulin levels. No signs of ketosis or drug-drug interaction were observed when metformin and dapagliflozin was administered with alpelisib. Alpelisib antitumor efficacy was maintained when used with dapagliflozin in tumor-bearing rats. Compared with a matched set of patients without SGLT2i, patients receiving SGLT2i had 4.9 and 6.4 times lower rates of grade ≥ 3 hyperglycemia AEs and hyperglycemia AEs resulting in alpelisib dose adjustments, interruptions, or withdrawals, respectively, and a relative reduction in risk of experiencing these AEs (70.6% and 35.7%).

**Conclusion:**

These data suggest adding an SGLT2i can effectively manage hyperglycemia, resulting in fewer alpelisib dose modifications and discontinuations in patients with *PIK3CA*-mutated HR+ /HER2− ABC (SOLAR-1: NCT02437318; BYLieve: NCT03056755).

**Supplementary Information:**

The online version contains supplementary material available at 10.1007/s10549-024-07405-8.

## Introduction

Breast cancer is the most commonly diagnosed cancer (24.5%) among women worldwide [1]. Patients with the hormone receptor–positive (HR+), human epidermal growth factor receptor 2–negative (HER2−) molecular subtype are known to be the largest subpopulation of breast cancer patients (> 70%) [2]. Currently, endocrine therapy (ET) combined with a cyclin-dependent kinase 4/6 inhibitor (CDK4/6i) is the standard of care in the first-line setting for patients with HR + /HER2 − advanced breast cancer (ABC) [3]. However, ≃ 40% of patients with HR + /HER2 − breast cancer have mutations in the *PIK3CA* gene, which can lead to resistance to endocrine-based therapy and eventual disease progression or relapse over time [4–8].

Alpelisib, an α-selective PI3K inhibitor and degrader, in combination with fulvestrant has demonstrated a significant progression-free survival (PFS) benefit over fulvestrant alone (hazard ratio, 0.65; 95% CI, 0.50–0.85; *P* < 0.001) in patients with *PIK3CA*-mutated HR+ /HER2− ABC that progressed on or after a previous aromatase inhibitor (AI) in the phase III SOLAR-1 trial (NCT02437318) [9]. Based on these results, alpelisib plus fulvestrant was approved for the treatment of this patient population following progression on or after ET [10–12]. Additionally, the phase II BYLieve trial (NCT03056755) reported that alpelisib plus fulvestrant showed activity in patients with *PIK3CA-*mutated HR+ /HER2− ABC immediately after disease progression on a CDK4/6i plus an AI (cohort A: median PFS, 7.3 months; 95% CI, 5.6–8.3 months), the most common scenario for alpelisib exposure in a real-world clinical setting [13].

The on-target effects of inhibition of the PI3Kα pathway include adverse events (AEs) such as hyperglycemia, diarrhea, and rash [9, 14]. Specifically, α-selective PI3K inhibition can lead to hyperglycemia through blocking of glucose uptake by skeletal muscle and adipose tissue and by activation of hepatic glyconeogenesis [9, 14]. Furthermore, unchecked hyperglycemia can lead to life-threatening complications, including diabetic ketoacidosis and hyperglycemic hyperosmolar syndrome [15]. In SOLAR-1, 63.7% of patients in the alpelisib arm had hyperglycemia (all grades), and 6.3% discontinued treatment due to hyperglycemia. Additionally, high rates of alpelisib-associated hyperglycemia (80.3%) have been observed in a real-world setting [9, 16]. Thus, hyperglycemia management is crucial in these patients to enable them to continue on alpelisib.

To manage hyperglycemia in SOLAR-1, antihyperglycemic medication, most commonly metformin in 87.1% of patients with hyperglycemia, was used and the protocol amended to include additional AE management guidelines [9, 14]. Using this information, hyperglycemia was supervised and managed more successfully in BYLieve, resulting in a lower incidence of all-grade hyperglycemia (58%) and fewer treatment discontinuations due to hyperglycemia (2%) [13]. However, an unmet need remains for management strategies for patients treated with alpelisib that offer earlier and more lasting improvement of hyperglycemia than what is currently achieved.

Sodium glucose cotransporter 2 inhibitors (SGLT2is) are a relatively new class of antihyperglycemic medications used for managing hyperglycemia in patients with type 2 diabetes. These drugs reduce glucose renal reabsorption and facilitate its excretion, thus reducing blood glucose levels [17]. Interestingly, a study in mice found that SGLT2 inhibition reduces PI3K inhibition–induced increase in blood glucose and plasma C-peptide levels [18]. Thus, in this analysis, we used data from preclinical experiments and two clinical trials to investigate the benefit of using an SGLT2i for the management of alpelisib-induced hyperglycemia.

### Methods

Preclinical experiments were performed on three rat strains to ascertain the extent of glucose and insulin control achievable with administration of alpelisib plus an SGLT2i (dapagliflozin) with or without metformin as well as the effects on alpelisib tolerability and antitumor efficacy. Clinical data from a subset of patients from the SOLAR-1 and BYLieve trials were analyzed. Specifically, this exploratory analysis examined grade ≥ 3 hyperglycemia AEs and hyperglycemia AEs resulting in dose reductions, drug interruptions, or drug withdrawals in patients who received or did not receive an SGLT2i plus alpelisib in SOLAR-1 and BYLieve. The Common Terminology Criteria for Adverse Events (CTCAE) version 4.03 was used to assess hyperglycemia in both trials.

### Animal studies

Three rat strains (Charles River Laboratories, Germany) were used: 10- to 12-week-old healthy female Brown Norway (BN) rats, 8- to 11-week-old mild diabetic male Zucker diabetic fatty (ZDF) rats mild diabetic male Zucker diabetic fatty (ZDF) rats, and Rat1-myr-p110α and HBRX3077 patient-derived estrogen receptor (ER)–positive/*PIK3CA*-mutated tumor-bearing nude rats. Rat1-myr-HA-p110α tumors were established by subcutaneous injection of 3 × 10^6^ cells in 200.0 μL of HBSS (Sigma H8264) in Matrigel (50%:50%) into the right flank of the nude rats. HBRX3077 patient-derived ER-positive/*PIK3CA*-mutated xenograft tumors were passaged by serial transplant into the right flank of the nude rats. All animals had access to food and water ad libitum over the course of the experiments.

Blood glucose levels in BN and ZDF rats were continuously measured for 14 days via a 1.4-cc telemetry device surgically implanted into their intraperitoneal cavity, with glucose sensors in the abdominal aorta and data collected on the Dataquest A.R.T. acquisition system (Data Sciences International). For some experiments, plasma insulin and glucose levels were measured using a commercially available ELISA kit (Mercodia) and a glucometer (OneTouch® Ultra®, LifeScan), respectively.

Tumor volume and body weight of tumor-bearing nude rats were measured two to three times per week. Tumor size was measured with calipers, and tumor volume was estimated using the formula (width × height × length) × π/6, with these measurements being the three largest diameters. Treatments were initiated when the mean tumor volume in each group reached 1000–1500 mm^3^.

Alpelisib, dapagliflozin, and metformin were formulated in 1% carboxymethylcellulose plus 0.5% Tween 80 and dosed at 5.0 mL/kg via oral gavage. Alpelisib as a single agent, alpelisib/metformin and alpelisib/dapagliflozin as double combinations, and alpelisib/metformin/dapagliflozin as a triple combination were compared for changes in plasma glucose levels, insulin levels, ketone body levels, blood lactate levels, body weight, and drug-drug interactions.

### Clinical trials data

#### Study designs

For the SOLAR-1 trial, postmenopausal women or men with HR+ /HER2− ABC that progressed on or after treatment with an AI were enrolled into the *PIK3CA*-mutated or *PIK3CA-*nonmutated cohort [9]. For the BYLieve trial, women and men aged ≥ 18 years with HR+ /HER2− ABC not amenable to curative therapy and with a confirmed *PIK3CA* mutation were enrolled into cohorts A, B, and C based on immediate prior line of therapy: CDK4/6i plus an AI, CDK4/6i plus fulvestrant, or chemotherapy or ET, respectively. Details of the trial designs have been published previously [9, 13]. AEs were assessed at protocol-defined periods (Supplementary Methods).

#### Inclusion criteria for SOLAR-1 and BYLieve based on baseline plasma glucose levels

SOLAR-1 included patients with hemoglobin A1c (HbA1c) < 8% at the start of the trial; however, based on recommendations by an advisory board of experts in AE management, this was later modified to < 6.5% to exclude patients with uncontrolled diabetes. Instruction on lifestyle modifications at screening and consultation with a healthcare specialist were recommended for patients with baseline fasting plasma glucose (FPG) ≥ 5.6 mmol/L and/or HbA1c ≥ 5.7% [9, 14]. For BYLieve, patients needed to have an FPG ≤ 7.7 mmol/L and HbA1c ≤ 6.4%. For both trials, baseline glycemic status was defined according to the American Diabetes Association: normal (FPG < 5.6 mmol/L and HbA1c < 5.7%), prediabetic (FPG ≥ 5.6 to < 7.0 mmol/L and/or HbA1c ≥ 5.7% to < 6.5%), and diabetic (FPG ≥ 7.0 mmol/L and/or HbA1c ≥ 6.5%) [13, 14].

### Analysis in patients who received an SGLT2i (SGLT2i cohort) vs those who did not (control cohort)

#### Propensity score matching

Patients in the SGLT2i cohort included in this analysis were classified based on World Health Organization drug coding of A10BK (ATC code level 4). Any patient from SOLAR-1 who received alpelisib and an SGLT2i was included, irrespective of *PIK3CA* mutation status. Any patient from BYLieve who received alpelisib and an SGLT2i from all three cohorts was included.

Patients in the SGLT2i cohort were compared with a matched control cohort (control cohort) selected from the set of patients in the two trials who received alpelisib but not an SGLT2i (non-SGLT2i patient set). Specifically, each patient’s propensity score was obtained from a logistic regression model with SGLT2i treatment status regressed on the following four risk factors: age, body mass index (BMI), HbA1c, and FPG. Next, each SGLT2i patient was matched with patients from the non-SGLT2i set whose propensity scores were closest to the former’s propensity score via nearest neighbor matching. Non-SGLT2i patients with missing data in the four risk factors were excluded from the set.

Several measures were used to ensure quality of matching, including distribution of propensity scores among matched and unmatched patients, mean differences in the four risk factors, propensity scores for the SGLT2i cohort versus all patients and control patients, and median values and interquartile ranges for these risk factors between the SGLT2i and control cohorts (Supplementary Methods).

#### SGLT2i/placebo start dates

For each patient in the SGLT2i cohort, the date on which the patient was first administered an SGLT2i was noted as the SGLT2i treatment start date. For each of their matched controls in the control cohort, the start date for an artificial placebo (henceforth referred to as “placebo”) was constructed as described in the Supplementary Methods. Matched control patients who had a last exposure date that occurred before their placebo start date were excluded from the analysis; SGLT2i patients were rematched if necessary (Supplementary Methods).

#### Hyperglycemia AEs: incidence rates and risk analysis

Placebo start dates for the SGLT2i and matched control cohorts were used to compare incidence rates and time to the first grade ≥ 3 hyperglycemia AE and hyperglycemia AE that resulted in an alpelisib adjustment, interruption, or withdrawal following the start of SGLT2i treatment.

The incidence rate for the SGLT2i cohort $k\in \{\text{0,1}\}$ (SGLT2i cohort, $k=0;$ control cohort, $k=1$) was calculated as $I{R}_{k}=\frac{{\sum }_{i\in {I}_{k}} \text{Number of hyperglycemia AEs experienced by patient i }}{{\sum }_{i\in {I}_{k}} \text{Exposure duration for patient i}}$

where ${I}_{k}$ is the set of patients in cohort $k$. Exposure duration was defined as the length of the patient’s exposure period, i.e., the duration between their SGLT2i/placebo start date and last exposure date (up to 30 days following their last dose of study treatment). Therefore, the incidence rate for treatment cohort $k$ was calculated as the total number of hyperglycemia AEs (either grade ≥ 3 or leading to alpelisib dose modifications/discontinuations) experienced by the patients in cohort $k$ during their exposure periods, divided by the sum of exposure durations for patients in cohort $k$.

Time to the first grade ≥ 3 hyperglycemia AE and first hyperglycemia AE leading to alpelisib adjustment, interruption, or discontinuation was estimated using the Kaplan–Meier method. The hazard ratios for these outputs were estimated using a Cox proportional hazards model with SGLT2i treatment as a covariate.

## Results

### Results from rat models

#### BN rats

**Fig. 1:**
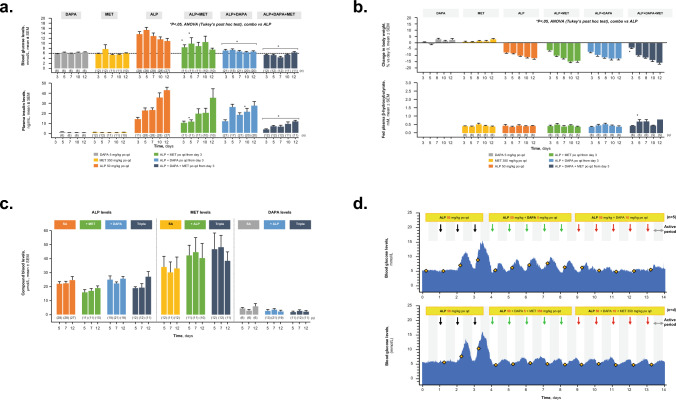
BN rats treated with DAPA, MET, or ALP, double combination of ALP + MET or ALP + DAPA, or triple combination of ALP + DAPA + MET. **a** Blood glucose and plasma insulin levels. **b** Change in body weight and fed plasma β-hydroxybutyrate. **c** Study drug levels. **d** Blood glucose levels in BN rats treated sequentially with ALP combined with increasing dose of MET (n = 5) or ALP combined with MET and increasing doses of DAPA (n = 4). *ALP* alpelisib, *ANOVA* analysis of variance, *BN* Brown Norway, *combo* combination, *DAPA* dapagliflozin, *MET* metformin, *po* orally, *qd* once daily, *SA* single agent

In BN rats, alpelisib administered as a single agent induced hyperglycemia and hyperinsulinemia. Addition of dapagliflozin to alpelisib suppressed alpelisib-induced hyperglycemia and slightly reduced insulin levels (Fig. 1a). The triple combination of alpelisib plus metformin and dapagliflozin was the most effective in normalizing blood glucose and insulin levels compared with the double combinations.

Furthermore, the triple combination of alpelisib with metformin and dapagliflozin did not impact alpelisib-induced body weight loss in BN rats, and they showed no signs of ketosis (Fig. 1b). Interestingly, the combinations that included metformin led to slightly greater alpelisib-induced body weight loss in these rats. Finally, no signs of drug-drug interactions were observed with any of the combinations compared with a single agent (Fig. 1c).

Experiments that continuously measured changes in blood glucose levels over 14 days in the same BN rats were also conducted. These experiments showed that alpelisib 50 mg/kg orally (po) once daily (qd) induced hyperglycemia by the third day of administration (Fig. 1d). Combining increasing doses of dapagliflozin (5 and 10 mg/kg po qd) with alpelisib significantly reduced blood glucose levels over 24-h dosing periods. In a different set of BN rats, blood glucose levels showed a rapid onset and sustained normalization when metformin (350 mg/kg po qd) and dapagliflozin (5 and 10 mg/kg po qd) were combined with alpelisib following alpelisib-induced hyperglycemia.

#### ZDF rats

**Fig. 2:**
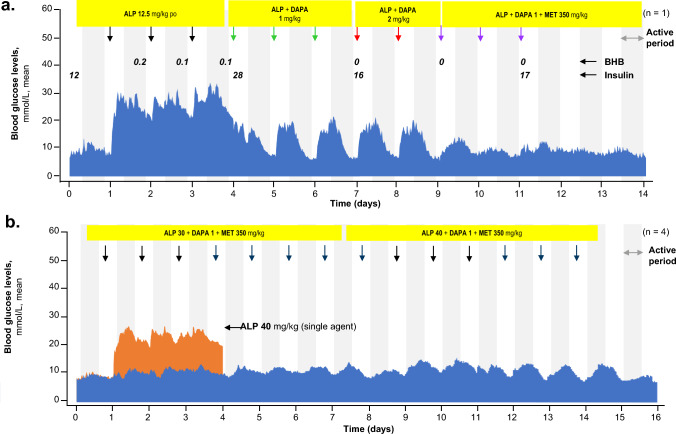
Blood glucose levels in ZDF rats treated sequentially with 12.5 mg/kg (**A**) and 30 and 40 mg/kg (**B**) dose of ALP combined with DAPA + MET. *ALP* alpelisib, *BHB* β-hydroxybutyrate, *DAPA* dapagliflozin, *MET* metformin, *po* orally, *ZDF* Zucker diabetic fatty

The diabetic status of ZDF rats was confirmed using a glucose tolerance test (Supplementary Fig. 1). Repeated daily doses of dapagliflozin (1 and 2 mg/kg) plus metformin (350 mg/kg) with alpelisib (12.5 mg/kg) normalized alpelisib-induced increases in blood glucose levels over 24-h dosing periods (Fig. 2a). Hyperinsulinemia was observed at 4 h post treatment, but no increase in ketone body β-hydroxybutyrate (BHB) was observed. When alpelisib was administered at 40 mg/kg as a single agent, it induced severe hyperglycemia in the ZDF rats (Fig. 2b). Dapagliflozin and metformin were efficacious at reducing blood glucose levels even with higher doses of alpelisib (30 and 40 mg/kg).

**Fig. 3:**
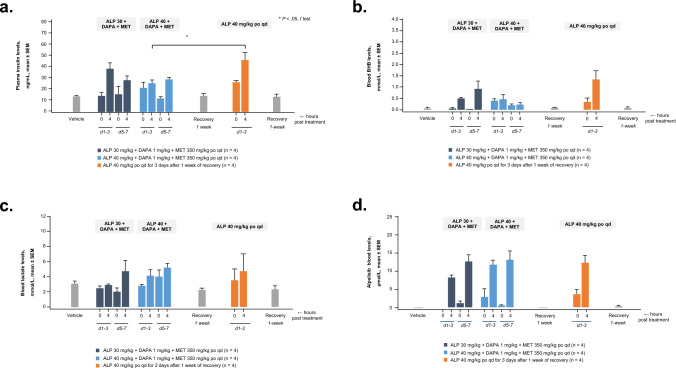
Plasma insulin (**a**), ketone (**b**), lactate (**c**), and ALP (**d**) levels in blood of ZDF rats treated with ALP, double combination of ALP + MET, or triple combination of ALP + DAPA + MET. *ALP* alpelisib, *BHB* β-hydroxybutyrate, *d* day, *DAPA* dapagliflozin, *MET* metformin, *po* orally, *qd* once daily, *ZDF* Zucker diabetic fatty

Plasma insulin levels increased in ZDF rats following alpelisib (40 mg/kg) administration as a single agent (Fig. 3a). When alpelisib (30 and 40 mg/kg) was administered with dapagliflozin and metformin, levels of plasma insulin were significantly reduced. Alpelisib administered alone at 40 mg/kg induced a mild increase in ketone body BHB levels at 4 h post treatment (Fig. 3b). When alpelisib was combined with dapagliflozin and metformin, a smaller increase in BHB levels was observed. Furthermore, blood lactate levels did not change when alpelisib was administered alone or with metformin and dapagliflozin (Fig. 3c). Finally, when alpelisib was combined with dapagliflozin and metformin, alpelisib blood levels at 4 h post treatment remained similar to those of alpelisib alone in ZDF rats (Fig. 3d).

#### Tumor-bearing rats

**Fig. 4:**
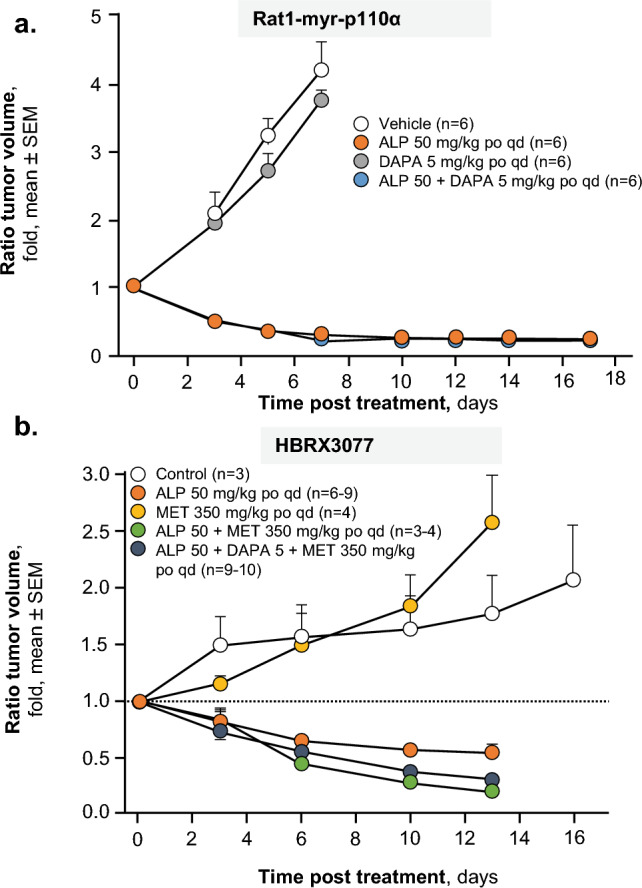
Antitumor efficacy of ALP or DAPA, double combination of ALP + DAPA, or triple combination of ALP + DAPA + MET in Rat1-myr-p110α (**a**) and HBRX3077 patient-derived (**b**) *PIK3CA*-mutated tumor-bearing nude rats. *ALP* alpelisib, *DAPA* dapagliflozin, *MET* metformin, *po* orally, *qd* once daily

The antitumor efficacy (measured by reduction in tumor size) of alpelisib was maintained when alpelisib was combined with dapagliflozin in Rat1-myr-p110α rats (Fig. 4). The efficacy of alpelisib in reducing tumor volume was slightly improved (not statistically significant) when alpelisib was combined with metformin and dapagliflozin in ER+/*PIK3CA* mutant HBRX3077 PDX tumor-bearing nude rats**.** Improvements in alpelisib-induced hyperglycemia and hyperinsulinemia similar to those seen in BN rats were observed when dapagliflozin (with or without metformin) was added to alpelisib in tumor-bearing nude rats.

### Patient data from the SOLAR-1 and BYLieve trials

#### Demographics and baseline characteristics in the SGLT2i and control cohorts

For this analysis, the data cutoffs were 23 April 2020 (SOLAR-1), 14 June 2021 (BYLieve cohort A), 14 August 2020 (BYLieve cohort B), and 14 June 2021 (BYLieve cohort C). All patients in the SGLT2i cohort (N = 19) were female; two were premenopausal and seventeen were postmenopausal. Overall, 16 of 19 patients (84.2%) had a *PIK3CA* mutation (10 from BYLieve and six from SOLAR-1). Median age of the patients was 62.0 years (quartile [Q] 1-Q3, 55.5–67.0 years); one patient (5.3%) was ≥ 75 years of age. Nine patients were Asian, eight were White; one was categorized as other race, and one was missing race data. Mean BMI (n = 18) was 27.9 kg/m^2^ (Q1-Q3, 24.4–29.0 kg/m^2^); eight patients (42.1%) were classified as overweight (BMI, 25–29.9 kg/m^2^) and four (21.1%) were classified as obese (BMI, ≥ 30 kg/m^2^). BMI data were missing for one patient. Three patients (15.8%) were diabetic (HbA1c ≥ 6.5% or FPG ≥ 126 mg/dL), 14 (73.7%) were prediabetic (HbA1c ≥ 5.7% to < 6.5% or FPG ≥ 100 to < 126 mg/dL), and two (10.5%) were in the normoglycemic range (HbA1c < 5.7% or FPG < 100 mg/dL). Baseline characteristics of patients in the control cohort were comparable to those of patients in the SGLT2i cohort (Supplementary Table 1).

#### Patient disposition

In the SGLT2i cohort, five of 19 patients (26.3%) were still receiving study treatment. Most patients (n = 13 [68.4%]) had discontinued treatment due to progressive disease at the time of data cutoff. None of these patients entered post-treatment follow-up. One patient died during the study. No patients in this cohort discontinued alpelisib due to a hyperglycemia event.

In the control cohort, 12 of 74 patients (16.2%) were still receiving study treatment. Most patients (n = 50 [67.5%]) had discontinued treatment due to progressive disease at the time of data cutoff. Three discontinued due to an AE, eight discontinued for other reasons, and one patient died during the study.

#### Antihyperglycemic medications received during the trial

All 19 patients (100%) in the SGLT2i cohort and 50 of 74 patients (62.2%) in the control cohort received an anti-diabetes medication, such as metformin, at some point during alpelisib treatment (Supplementary Table 2).

#### Duration of exposure to alpelisib and SGLT2i

The median duration of alpelisib treatment (N = 19) in the SGLT2i cohort was 10.2 months (Q1-Q3, 5.5–18.3 months). As SGLT2is were started late in the treatment plan, the median duration of SGLT2i treatment (derived from n = 16 patients; three had ongoing SGLT2i treatment and were excluded) was shorter (4.7 months [Q1-Q3, 2.5–12.5 months]), with most patients (n = 10 [52.6%]) being exposed to an SGLT2i for ≤ 6 months during the study. The median duration of alpelisib treatment (N = 74) in the control cohort was 9.7 months (Q1-Q3, 4.5–18.5 months).

#### *Grade* ≥ *3 hyperglycemia AEs*

**Fig. 5:**
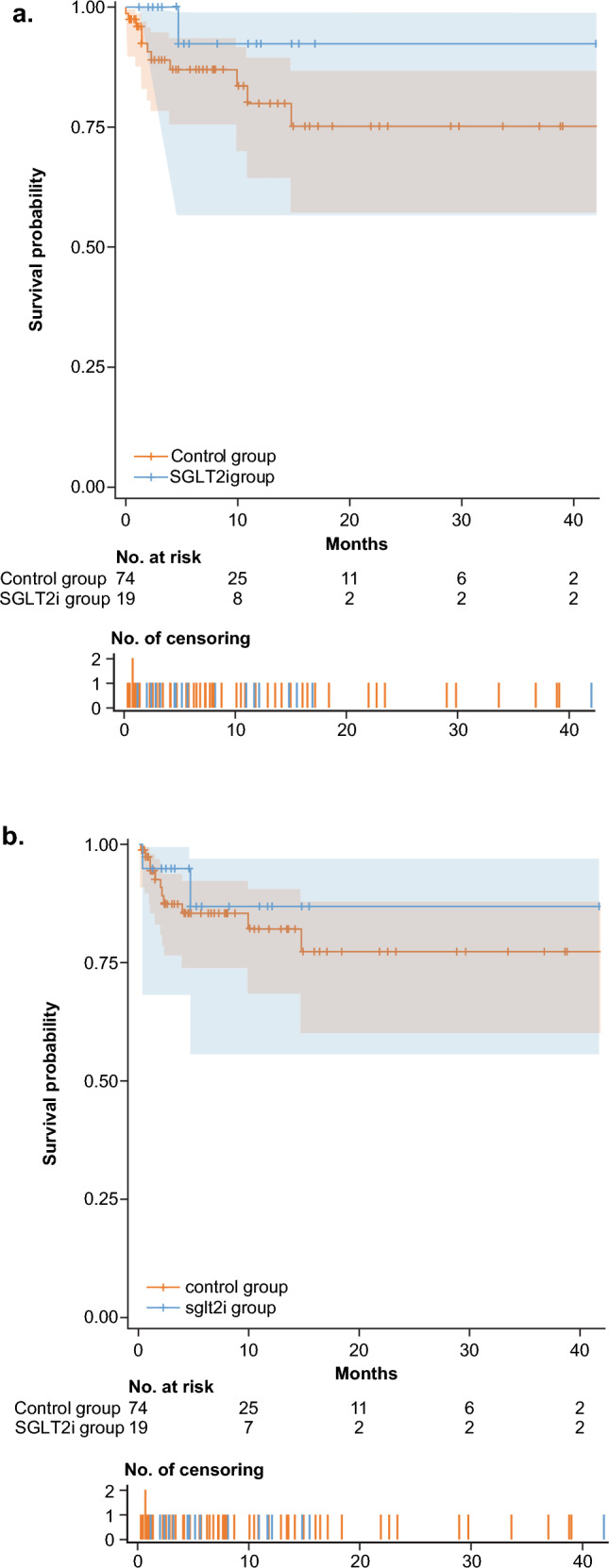
Time to first grade ≥ 3 hyperglycemia AE (**a**) and time to first hyperglycemia AE that led to dose adjustments, drug interruptions, or drug withdrawals (**b**) in patients in the SGLT2i and control cohorts. *AE* adverse event, *SGLT2i* sodium glucose cotransporter 2 inhibitor

For the SGLT2i cohort, the grade ≥ 3 hyperglycemia AE incidence rate was 0.00461 (one total grade ≥ 3 hyperglycemia AE and total exposure duration of 217 months). For the control cohort, the incidence rate was 0.02272 (18 total grade ≥ 3 hyperglycemia AEs and total exposure duration of 792 months). Thus, patients in the SGLT2i cohort had a 4.9 times lower incidence rate of grade ≥ 3 hyperglycemia AEs than patients in the control cohort. Analysis of time to the first grade ≥ 3 hyperglycemia AE demonstrated that addition of an SGLT2i to the treatment regimen resulted in a 70.6% relative reduction in the risk of experiencing a grade ≥ 3 hyperglycemia AE compared with the control cohort (hazard ratio, 0.294; Fig. 5a).

#### Hyperglycemia AEs that led to alpelisib adjustment, interruption, or withdrawal

For the SGLT2i cohort, the incidence rate of hyperglycemia AEs that resulted in dose adjustment, drug interruption, or drug withdrawal was 0.00922 (two incidents and total exposure duration of 217 months). For the control cohort, the incidence rate was 0.05917 (50 incidents and total exposure duration of 845 months). Thus, patients in the SGLT2i cohort had a 6.4 times lower incidence rate of hyperglycemia AEs that led to dose adjustment, drug interruption, or drug withdrawal than patients in the control cohort. Analysis of time to the first event (hyperglycemia AE leading to dose adjustment, drug interruption, or drug withdrawal) demonstrated that addition of an SGLT2i to the treatment regimen resulted in a 35.7% relative reduction in risk (hazard ratio, 0.643; Fig. 5b).

## Discussion

Overall, the preclinical and clinical data reported here suggest that the use of an SGLT2i may decrease the incidence of subsequent hyperglycemia events. Data from healthy BN rats and from mild diabetic ZDF rats, which model a patient population at risk for developing alpelisib-induced hyperglycemia, showed that the addition of metformin and dapagliflozin to alpelisib was the most effective treatment in preventing alpelisib-induced hyperglycemia and hyperinsulinemia. These rat models showed no indications of drug-drug interactions and no signs of euglycemic diabetic ketoacidosis; however, there is a risk of euglycemic diabetic ketoacidosis with SGLT2i treatment and regular monitoring is still required [19]. Importantly, the dose of alpelisib administered was similar to the clinically relevant dose for rats (12.5–25.0 mg/kg). Experiments on tumor-bearing nude rats showed that when alpelisib was combined with dapagliflozin and metformin, its antitumor efficacy was maintained. In the data analyzed from SOLAR-1 and BYLieve, patients receiving an SGLT2i had lower rates of alpelisib-induced grade ≥ 3 hyperglycemia AEs and hyperglycemia AEs that led to alpelisib dose adjustments, interruptions, or withdrawals; they also had a reduced risk of experiencing these AEs compared with patients with a similar hyperglycemia risk who did not receive an SGLT2i. This suggests that SGLT2i use may help reduce the frequency and severity of PI3Kα inhibitor–associated hyperglycemia AEs, and thus patients would require fewer dose modifications.

It has been shown that implementing a more detailed AE management plan improves hyperglycemia management during alpelisib treatment. In SOLAR-1, hyperglycemia AEs associated with alpelisib occurred relatively early during treatment and were reversible and manageable with monitoring and intervention, which included early identification of grade ≥ 3 hyperglycemia and administration of metformin [14]. The SOLAR-1 protocol was also amended to include a more stringent HbA1c inclusion criterion (< 6.5%) and training for investigators on supportive treatments [14]. These strategies informed AE management in BYLieve, resulting in a lower frequency of hyperglycemia-related discontinuations [13]. Also, patient risk factors that may predict a higher risk for grade ≥ 3 alpelisib-induced transient hyperglycemia have been identified, including baseline FPG, BMI, HbA1c, and age [20]. Addition of an SGLT2i to the treatment plan and identification of risk factors may lead to more effective hyperglycemia management during alpelisib treatment. Finally, clinical trials exploring diet and lifestyle changes along with administration of an SGLT2i for the management of alpelisib-associated hyperglycemia are also ongoing [21].

Many studies have shown the benefit of using SGLT2is for treating hyperglycemia in type 2 diabetes and its related complications, which has led to their approval by the US Food and Drug Administration for adults with type 2 diabetes [22–25]. The preclinical experiments show that the most effective combination to limit alpelisib-induced hyperglycemia AEs is an SGLT2i with metformin. Corroborating these preclinical data, the METALLICA trial has shown that prophylactic metformin use is effective in lowering the incidence of alpelisib-induced grade ≥ 3 hyperglycemia in patients with normal blood glucose and those who are prediabetic [26]. The current analysis comparing patients treated or not treated with an SGLT2i also support the preclinical data on SGLT2i use. While there is an imbalance in the proportion of patients who received an anti-diabetes medication between the SGLT2i and control cohort, it is important to note that each of the 19 SGLT2i patients was matched to at least one control patient who also received an anti-diabetes medication prior to (control) treatment. Based on these preclinical and clinical data, an investigation of the sequencing of antihyperglycemic agents is needed to fully understand the impact of SGLT2i use with other antihyperglycemic medications in managing patients treated with alpelisib.

## Conclusion

This study shows the effectiveness of SGLT2i treatment and supports its use in combination with or without metformin to manage hyperglycemia events in patients with HR+ /HER2− ABC treated with alpelisib, allowing patients to continue on treatment with fewer dose modifications.

## Supplementary Information

Below is the link to the electronic supplementary material.

Supplementary file1 (DOCX 317 KB)

## Data Availability

Novartis is committed to sharing with qualified external researchers, access to patient-level data and supporting clinical documents from eligible studies. These requests are reviewed and approved by an independent review panel on the basis of scientific merit. All data provided are anonymized to respect the privacy of patients who have participated in the trial in line with applicable laws and regulations.

## Declarations

### Research involving human and animal participants

The animal research performed in this study complies with all ethical regulations. Experiments were conducted under license number BS-2741 approved by the Cantonal Veterinary Office of Basel Stadt. Novartis is an Association for Assessment and Accreditation of Laboratory Animal Care–accredited facility.
