# Supplementary material for: SGLT2 inhibition improves PI3Kα inhibitor–induced hyperglycemia: findings from preclinical animal models and from patients in the BYLieve and SOLAR-1 trials
Source: Breast Cancer Res Treat. 2024 Aug 23;208(1):111–21. doi: 10.1007/s10549-024-07405-8 (PMC11452482; doi:10.1007/s10549-024-07405-8)
Supplement: Supplementary file 1 — Supplementary file1 (DOCX 317 KB) [file 10549_2024_7405_MOESM1_ESM.docx]

**Supplementary Information**

**Supplementary Fig. 1** Glucose tolerance test in BN and ZDF rats. A greater increase in plasma glucose was observed following an intraperitoneal dextrose injection (1.2 g/mL, 0.4 mL per rat) in ZDF rats compared with healthy BN rats, confirming the type 2 diabetes stage in these rats. BN, Brown Norway; ZDF, Zucker diabetic fatty


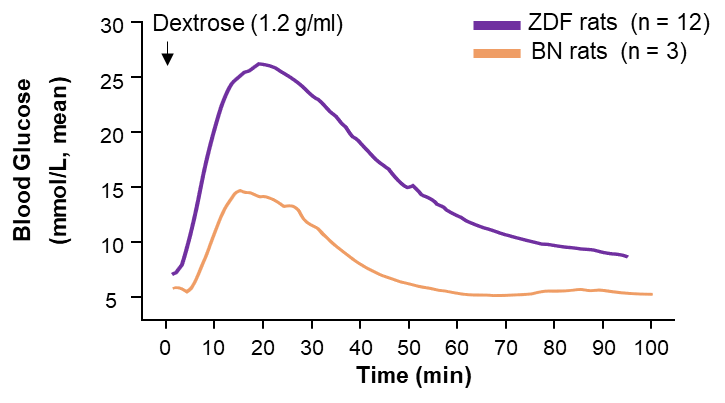


**Supplementary Table 1** Baseline characteristics of patients in the SGLT2i and control cohorts

| **Characteristics** | **SGLT2i cohort  (N=19)^a^** | **Control cohort**  **(N=74)** |
| --- | --- | --- |
| Median age (Q1-Q3), ­years^b^ | 62.0 (55.5-67.0) | 60.0 (55.0-65.0) |
|  |  |  |
| Female, n (%)^c^ | 19 (100) | 73 (98.6) |
| Menopausal status, n (%) | | |
| Premenopausal | 2 (10.5) | 3 (4.1) |
| Postmenopausal | 17 (89.5) | 70 (94.6) |
| Race |  |  |
| Asian | 9 (47.4) | 19 (25.7) |
| White | 8 (42.1) | 45 (60.8) |
| Other | 1 (0.9) | 6 (8.1) |
| Unknown | 1 (0.9) | 4 (5.4) |
| Mean BMI (Q1-Q3), ­kg/m^2^ | 27.9 (24.4-29.0) | 27.8 (23.7-31.4) |
| BMI <25, n (%) | 6 (31.6) | 24 (32.4) |
| BMI 25-29.9, n (%) | 8 (42.1) | 27 (36.5) |
| BMI ≥30, n (%) | 4 (21.1) | 23 (31.1) |
| Unknown, n (%) | 1 (0.9) | 0 |
| Blood glucose levels, n (%) |  |  |
| HbA1c <5.7% or FPG <100 mg/dL | 2 (10.5) | 9 (12.2) |
| HbA1c ≥5.7% to <6.5% or FPG ≥100 to <126 mg/dL | 14 (73.7) | 52 (70.3) |
| HbA1c ≥6.5% or FPG ≥126 mg/dL | 3 (15.8) | 13 (17.6) |

BMI, body mass index; FPG, fasting plasma glucose; HbA1c, hemoglobin A1c; Q, quartile; SGLT2i, sodium glucose cotransporter 2 inhibitor

^a^Ten patients from the BYLieve trial and nine patients from the SOLAR-1 trial were included in the SGLT2i cohort

^b^One patient in the SGLT2i cohort and five patients in the control cohort were ≥75 years of age

^c^One patient in the control cohort was male

**Supplementary Table 2** Antihyperglycemic agents received by patients in the SGLT2i and control cohorts prior to alpelisib treatment start date and after the start of alpelisib treatment but prior to SGLT2i/placebo treatment start date

|  | SGLT2i cohort | | Control cohort |
| --- | --- | --- | --- |
| Prior to alpelisib treatment start date | Glibenclamide, glimepiride, linagliptin, metformin, metformin hydrochloride, pioglitazone | Metformin, metformin hydrochloride, metformin hydrochloride with sitagliptin, sitagliptin phosphate | |
| Prior to SGLT2i treatment start date (following start of alpelisib treatment) | Acarbose, glibenclamide, gliclazide, glimepiride, insulin, insulin aspart, insulin aspart protamine, insulin aspart with insulin degludec, insulin glargine, insulin lispro, isophane insulin, jentadueto, liraglutide, metformin, metformin hydrochloride, pioglitazone, pioglitazone hydrochloride, repaglinide, sitagliptin, sitagliptin phosphate | Glibenclamide, glimepiride, glipizide, insulin, insulin aspart, insulin glargine, insulin human, insulin human injection (isophane), insulin human zinc suspension, insulin lispro, insulin porcine, isophane insulin, linagliptin, metformin, metformin hydrochloride, pioglitazone, pioglitazone hydrochloride, sitagliptin phosphate, teneligliptin hydrobromide | |

SGLT2i, sodium glucose cotransporter 2 inhibitor

***Supplementary Methods***

*AE assessment in SOLAR-1 and BYLieve*

For both trials, AEs were assessed at screening, during treatment, and up to 30 days following the last dose of study treatment according to the Common Terminology Criteria for Adverse Events version 4.03. FPG was assessed at baseline and at different time points during the two trials: (1) on days 8 and 15 in the first 4 weeks for the SOLAR-1 trial, and (2) on days 8 and 15 of cycle 1, days 1 and 15 of cycle 2, and day 1 of subsequent cycles for the BYLieve trial.

*Matching patients in the SGLT2i cohort*

The matched control cohort was obtained by using nearest neighbors on the propensity score to find five control patients who were most similar to each of the SGLT2i patients in four risk factors—age, BMI, HbA1c, and FPG—using Euclidean distance to measure similarity. The propensity scores were obtained via a logistic regression model with SGLT2i treatment regressed on the four risk factors. Matching resulted in no repeated control patients, i.e., no matched control was matched to multiple SGLT2i patients.

Distribution of propensity scores of different patients is shown in **Supplementary Methods Fig. 1**. “Unmatched Treated Units” corresponds to SGLT2i patients for whom no matched controls were found (all SGLT2i patients were matched to five control patients and thus there are no propensity score dots here). “Matched Treated Units” corresponds to SGLT2i patients for whom matched controls were found (propensity scores for all 19 are shown). “Matched Control Units” are the matched controls that were found (19×5=95; 5 control patients matched to each of the 19 SGLT2i patients). The distribution of their propensity scores roughly matches those of the SGLT2i patients shown in the row above; however, for some SGLT2i patients with outlying propensity scores (further to the right), matches tended to be more spread out, as those were the nearest (control) neighbors available to those SGLT2i patients. “Unmatched Control Units” are the control patients who did not match to any of the SGLT2i patients, and these were not considered in the analysis.


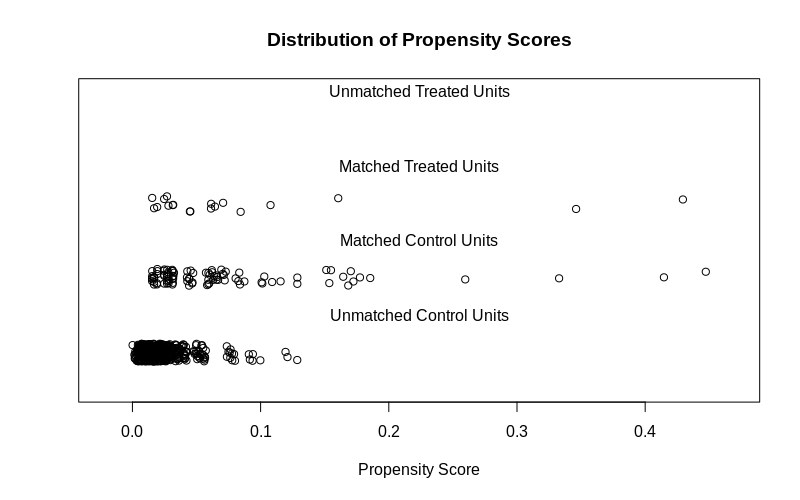
**Supplementary Methods Fig. 1** Distribution of propensity scores. Absolute standardized mean differences for each of the four risk factors (age, BMI, HbA1c, and FPG) and the propensity score is shown in **Supplementary Methods Fig. 2**. The absolute standardized mean difference is defined as the absolute value of the difference between the SGLT2i cohort’s mean and their matched control cohort’s or all patients’ mean, divided by the standard deviation calculated from all patients (matched and unmatched). The white dots represent the absolute standardized mean difference, calculated from all patients’ data for that variable. The black dots represent the absolute standardized mean difference, calculated from only the matched patients’ data for that variable. Matching improved all variables except age, for which the two cohorts were already similar.

**Supplementary Methods Fig. 2** Absolute standardized mean differences for the four risk factors used in matching and the propensity score. Comparison of box plots of the four risk factors between the SGLT2i cohort and the control cohort demonstrates the quality of the matching. Box plots before matching are shown in **Supplementary Methods Fig. 3a**. After matching, the box plots for the matched control patients and the SGLT2i patients are more similar (**Supplementary Methods Fig. 3b and 3c)**.


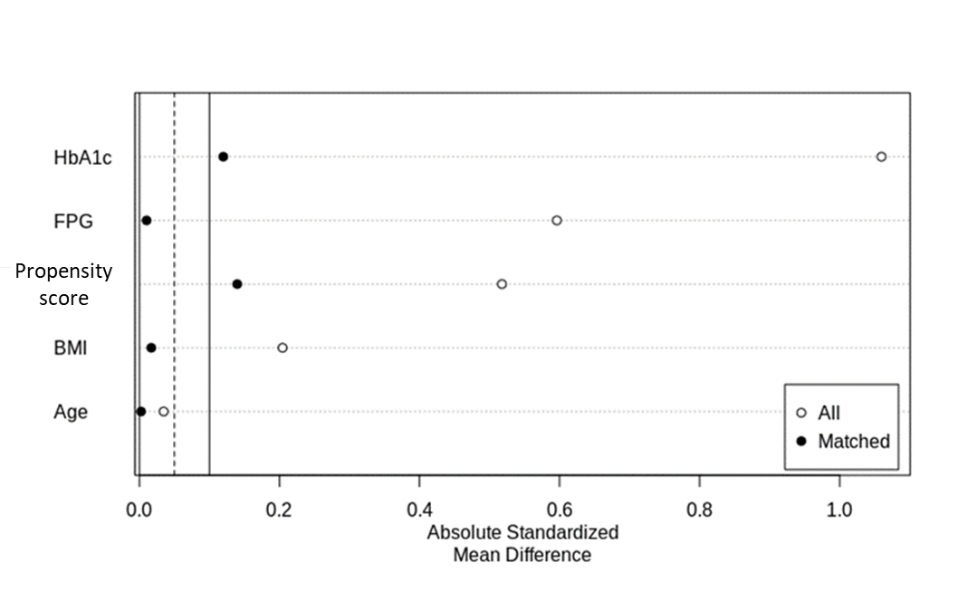
**Supplementary Methods Fig. 3** Box plot of the four risk factors for patients in the SGLT2i cohort and control cohort before (**a**) and after (**b**) matching. The aqua plots correspond to the 19 SGLT2i patients and the red plots correspond to all of the control patients who had no missing data (**a**), the originally matched 95 control patients (**b**), and the 74 control patients finally selected (**c**).

**a**


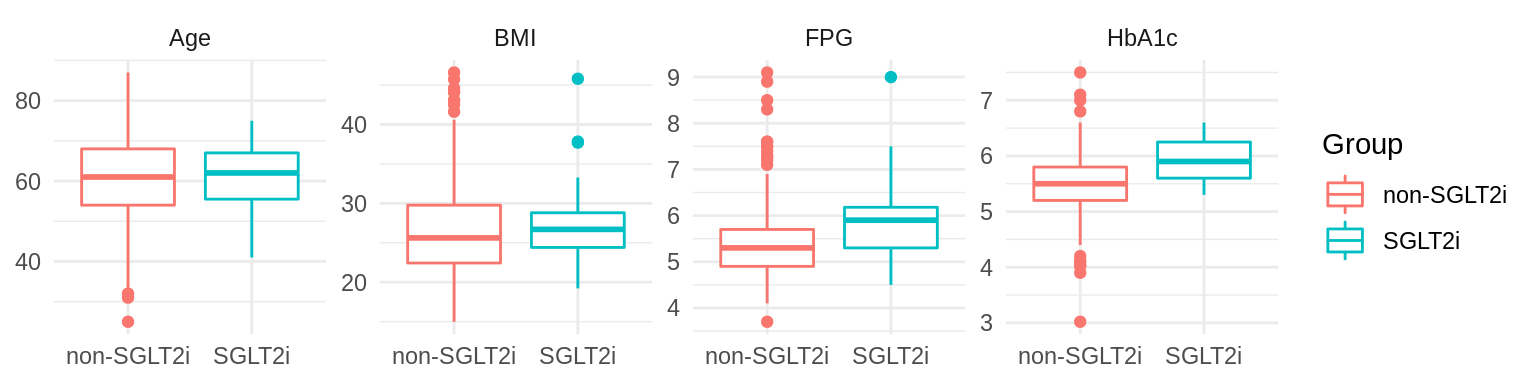


**b**


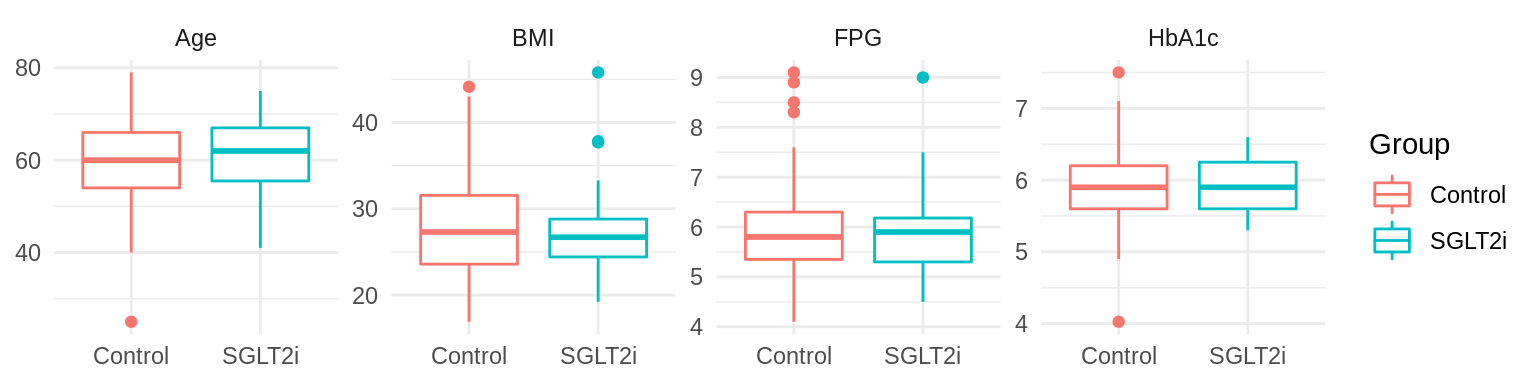


**c**


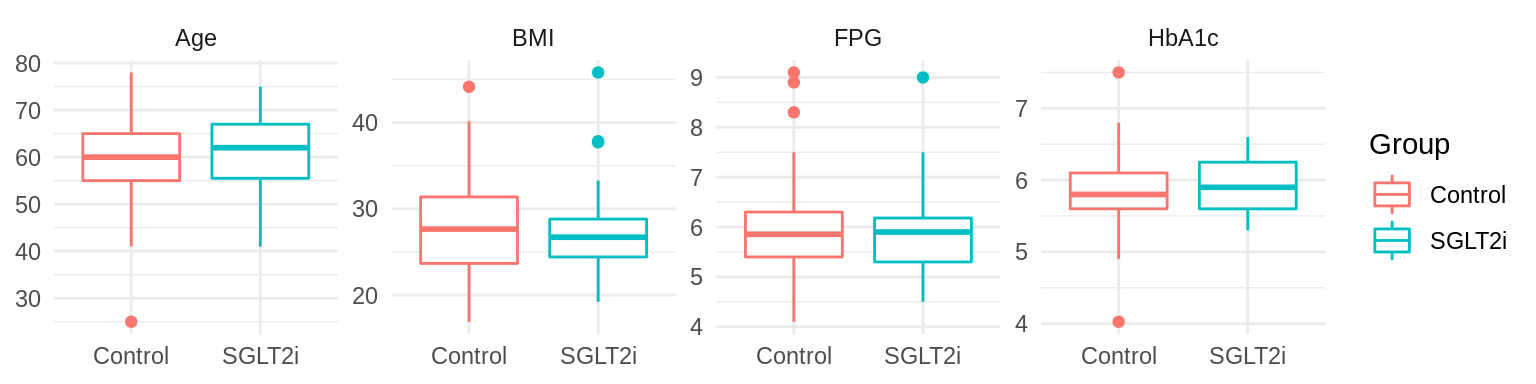


*SGLT2i/placebo start dates*

An artificial placebo date was constructed by calculating the duration between the SGLT2i patients’ alpelisib treatment start date and the SGLT2i start date and adding it to the alpelisib treatment start date for each of the respective control patients. Any control patient whose last exposure date for hyperglycemia AEs occurred before their placebo start date was removed from the analysis. This exposure date was determined by adding 30 days for safety follow-up to the date on which the final investigational treatment was received by the patient. If any patient in the SGLT2i cohort was left with no matches, the propensity score matching procedure was applied again to include more matches for these patients. For the final analysis, each SGLT2i patient had ≥1 matched control patient, and none of the control patients were matched to >1 SGLT2i patient (**Supplementary Methods Table 1**).

**Supplementary Methods Table 1** Number of matched control patients (control cohort) for each patient who received an SGLT2i

| ***SGLT2i patient*** | ***Total number of matched control patients, n*** |
| --- | --- |
| ***1*** | ***5*** |
| ***2*** | ***5*** |
| ***3*** | ***4*** |
| ***4*** | ***4*** |
| ***5*** | ***4*** |
| ***6*** | ***3*** |
| ***7*** | ***4*** |
| ***8*** | ***5*** |
| ***9*** | ***5*** |
| ***10*** | ***5*** |
| ***11*** | ***5*** |
| ***12*** | ***3*** |
| ***13*** | ***1*** |
| ***14*** | ***5*** |
| ***15*** | ***5*** |
| ***16*** | ***3*** |
| ***17*** | ***4*** |
| ***18*** | ***2*** |
| ***19*** | ***2*** |
